# Formulation and Evaluation of Controlled Release Floating Microballoons of Stavudine

**DOI:** 10.3797/scipharm.1501-07

**Published:** 2015-06-09

**Authors:** Suryadevara Vidyadhara, Reddyvalam Lankapalli Sasidhar, Talamanchi Balakrishna, Boyapati Balaji, Ravi Amrutha

**Affiliations:** Department of Pharmaceutics, Chebrolu Hanumaiah Institute of Pharmaceutical Sciencies, Chandramoulipuram, Chowdavaram, Guntur-522019, India

**Keywords:** Stavudine, Floating Microballoons, HPMC E_5_, Controlled Release, Eudragit RS 100

## Abstract

The aim of this study was to formulate and evaluate stavudine floating microballoons for controlled drug release. Initially, the drug-loaded low-density granular pellets were prepared with hydroxypropyl methylcellulose E5 grade and by using isopropyl alcohol as a granulating fluid. Further, the low-density granular pellets were subjected to microencapsulation by an emulsion evaporation technique using ethyl cellulose 7 cps and Eudragit S 100 as coating polymers and 1% w/v polyethylene glycol 400 as aqueous phase. The prepared microballoons were characterized for their particle size analysis, angle of repose, and compressibility index. The *in vitro* release studies were performed in 0.1 N HCl as medium. The prepared microballoons were free-flowing and spherical in shape. From all the formulations, F5E and F5F can be considered as promising controlled release floating microballoons of stavudine providing first-order release over a period of 12 hours, with a minimum floating lag time of 1 minute. It was found that the ratio of the drug & polymer, stirring speed, and concentration of surfactant were the most significant variables which influenced the size of the stavudine microballoons under the applied experimental conditions.

## Introduction

Drugs that have good absorption in the gastrointestinal tract (GIT) and a short half-life are eliminated quickly from systemic circulation. These types of drugs require frequent dosing. To overcome these problems, oral controlled release (CR) formulations have been developed. In this regard, controlled drug delivery systems have many benefits, which include improved therapy by increasing the efficacy and gastrointestinal transit time, increased patient compliance through decreased dosing frequency, convenient routes of administration, and improved site-specific delivery to reduce unwanted adverse effects [1, 2]. Gastro-retentive floating microspheres are low-density systems that have sufficient buoyancy to float over gastric contents and remain in the stomach for a prolonged period. As the system floats over gastric contents, the drug is released slowly at the desired rate resulting in increased gastric retention with reduced fluctuations in plasma drug concentration [3]. Microspheres can be defined as solid, spherical, empty particles ranging in size from 1 to 1000 µm [4]. Solid biodegradable microspheres incorporating a drug dispersed or dissolved throughout a particle matrix have the potential for the controlled release of a drug [5–7].

Floating drug delivery systems are designed with the preparation of a low-density solid system like sponges and floating microballoons or a system which decreases in density upon contact with gastric fluids based on the explosion of swelling agents or carbon dioxide generation. Multiple unit particulate dosage forms like microspheres and microballoons have the advantages that they pass uniformity throughout the GIT to avoid the vagaries of gastric emptying and provide an adjustable release, thereby reducing the inter-subject variability in absorption.

Among the various methods developed for the formulation of microballoons, the solvent evaporation method has gained much attention due to its ease of fabrication without compromising the activity of the drug [8]. Stavudine (D4T, thymidine) is the FDA-approved drug for clinical use for the treatment of HIV infection, AIDS, and AIDS-related conditions either alone or in combination with other antiviral agents. Stavudine is typically administered orally as a capsule and an oral solution. The virustatic drug has a very short half-life (1.30 h). However, patients receiving stavudine develop neuropathy and lactic acidosis. The side effects of stavudine are dose-dependent and a reduction of the total administered dose reduces the severity of the toxicity [9].

The objective of the present investigation was to formulate and evaluate stavudine microballoons for controlled release. Hence, different batches of microballoons were prepared according to the working plan. The resultant microballoons were evaluated for percentage yield, entrapment efficiency, particle size, and *in vitro* drug release. The effect of process variables on the microballoons was also studied.

## Experimental

### Materials and Reagents

Stavudine was a gift sample and was obtained from Aurbindo Pharmaceuticals, Hyderabad, and hydroxylpropyl methylcellulose (METHOCEL) E5 was a gift sample from Colorcon Asia Pvt. Ltd, Mumbai).

Eudragit RS 100, ethyl cellulose 7 cps, and isopropyl alcohol were used and all the reagents and solvents were of analytical grade satisfying pharmacopoeial standards.

### Preparation of Microballoons by the Solvent Evaporation Technique

The microballoons were prepared using the solvent evaporation technique. Initially, the drug-loaded HPMC E5 low-density granules (LDG) and the prepared granules were dispersed into the polymer solution (Eudragit RS 100, ethyl cellulose) in methanol and dichloromethane (1:1) and then sonicated. The resulting suspension was added dropwise into an aqueous solution of polyethylene glycol (1% w/v) at 70°C. The suspension was stirred at 1500 rpm using a mechanical stirrer for 2 hr. During the stirring process, the solvent was evaporated, leaving the solid discrete microballoons coated with the coating polymer into an aqueous solution of polyethylene glycol. The microballoons were separated by filtration and dried at room temperature in desiccators for 24 hr. Then the microballoons were further evaluated for their physical parameters such as weight uniformity. The formulation tables are shown in Tables 1 & 2.

**Tab. 1:**
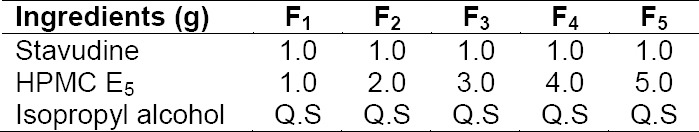
Composition of low-density granular pellets

**Tab. 2:**
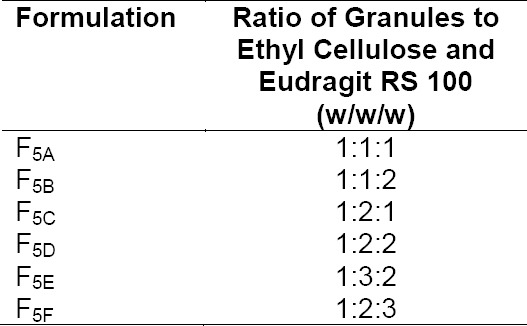
Composition of floating microballoons

### Characterization of Microballoons

#### Particle Size Analysis

Measurements of the particle size distribution of microspheres were carried out with an optical microscope. The stage micrometer was used to calculate the calibration factor. The particle size was calculated by multiplying the number of divisions of the ocular disc occupied by the particle with the calibration factor. Fifty randomly chosen spheres were taken to measure their individual size. The results are shown in Table 3.

**Tab. 3:**
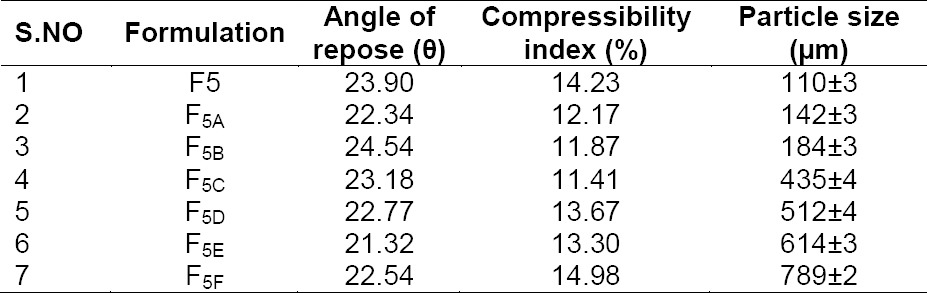
Physical evaluation of stavudine microballoons

#### Angle of Repose

Angle of repose of the microballoons, which measures the resistance to particle flow, was determined by the fixed funnel method. The angle of repose was calculated from the height and the average radius using the following formula:


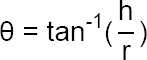


where θ = Angle of repose, h = Height of the pile, r = Average radius of the circle.

#### Compressibility Index

A simple test was used to evaluate the flowability of a powder by comparing the bulk density and the tapped density of a powder and the rate at which it is packed down. The Carr’s index was calculated using the following formula:


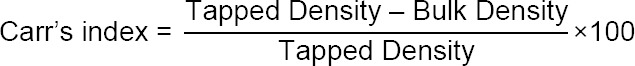


#### Drug Content

The drug content of the prepared microballoons was determined by dispersing 50.0 mg of the formulation in 10 ml of 95% ethanol followed by agitation with a magnetic stirrer up to 12 hr to dissolve the polymer and extract the drug. Then the solution was filtered through a 5 µm membrane filter and the drug concentration was determined spectrophotometrically at 240 nm using a double beam UV spectrophotometer.

The percentage drug entrapment was calculated by using the formula:


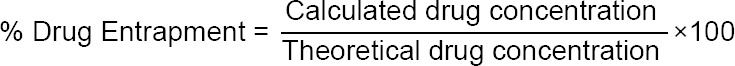


#### In Vitro Dissolution Studies

The dissolution test for the prepared microparticles was carried out in the USP Apparatus Type II (Paddle) [USPNF, 2007] employing 900 ml of 0.1 N HCl as the dissolution medium. Five ml of the samples were withdrawn at regular time intervals at 1, 2, 4, 6, 8, 10, and 12 hr. Fresh medium up to the volume was replaced with the withdrawn volume to maintain the sink conditions and constant volume throughout the experiment. Samples withdrawn were suitably diluted with the same dissolution medium and the amount of drug dissolved was estimated by the ELICO SL-210 Double Beam Spectrophotometer at 240 nm and the cumulative percentage of drug released was subsequently calculated.

#### In Vitro Buoyancy Studies

All of the prepared floating buoyancy study was characterized by floating lag time and total floating time. The test was performed using a USP Type II Paddle Apparatus using 900 ml of 0.1 N HCl at a paddle rotation of 50 rpm at 37 ± 0.5°C. The time required for the microballoons to rise to the surface of the dissolution medium and the duration of time the tablet constantly floated on the dissolution medium were noted as floating lag time and total floating time, respectively. The results are shown in Table 4.

**Tab. 4:**
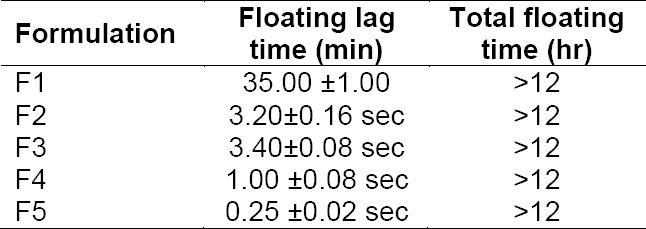
Floating lag times and total floating times of various microballoons of stavudine (mean ± S.D.; n=3)

#### Fourier Transform Infrared Spectroscopy (FTIR)

Drug-polymer interactions were studied by FTIR spectroscopy. The spectra were recorded for the pure drug and drug-loaded microballoons using FTIR (Model No. IR Presige-21, Shimadzu). Samples were prepared in KBr disks (2 mg sample in 200 mg KBr). The scanning range was 400–4000 cm^−1^ and the resolution was 4 [1/cm].

#### Differential Scanning Calorimetry

The DSC analyses of the pure drug and drug-loaded microballoons were carried out using the Shimadzu DSC 60 to evaluate any possible drug-polymer interaction. The analysis was performed at a rate 10.0°C/min from a 200°C to 300°C temperature range under nitrogen flow of 25 ml/min.

## Discussion

The spectrophotometric method used for the estimation of stavudine in the dissolution medium was found to be linear and reproducible. The standard calibration curve yielded a straight line, which shows that the drug follows Beer’s law in the concentration range of 2–10 μg/ml with an R^2^ value of 0.998. Reproducibility of the method was tested by analyzing six separately weighed samples of the drug. Thus, the method was found to be suitable for the estimation of stavudine in dissolution medium.

Initially, low-density granular pellets of stavudine were formulated employing HPMC E5 at various concentrations. The prepared low-density granular pellets were evaluated for floating lag time and i*n vitro* drug release. Among the prepared low-density granular pellets, formulation F5 containing the drug : HPMC E5 in the ratio of 1:5 showed a floating lag time of 15 sec and *in vitro* drug release up to 2 hr. This formulation was optimized for preparing microballoons employing Eudragit RS 100 & ethyl cellulose 7 cps as coating polymers and 1% w/v PEG 400 as the aqueous phase.

The floating microballoons were prepared by the emulsion solvent evaporation technique. A suspension of Eudragit S100, ethyl celluose, and LDG with ethanol and dichloromethane was poured into an agitated aqueous solution of polyethylene glycol. The ethanol rapidly partitioned into the external aqueous phase and the polymer precipitated around the dichloromethane droplets. The subsequent evaporation of the entrapped dichloromethane led to the formation of internal cavities within the microballoons. The incorporation of the drug-adsorbed HPMC E5 into the formulation may produce a porous structure within the microballoons. The ultrasonication produced drug-adsorbed HPMC E5 in a fine state of subdivision. A potential advantage of using large volumes of the external aqueous phase is the reduction in the required stirring times.

The solubility of dichloromethane in water is 1% w/v. Using a larger volume (400 to 500 ml), the diffusion of dichloromethane into the aqueous phase and hence solidification of the particles occurred faster as compared to 200 ml. Thus, particles could be separated after shorter stirring times. It was found that a saturated solution of the polymer produced smooth and high yield microballoons. The undissolved polymer produced irregular and rod-shaped particles. At 70°C, the polymer and the drug were co-precipitated and the shell was formed by the diffusion of ethanol into the aqueous solution and simultaneous evaporation of dichloromethane. A portion of the polymer solution aggregated in a fibre-like structure, as it solidified prior to forming droplets, or the transient droplets were broken before the solidification was complete. As ethanol quickly diffused out of the organic phase (polymer solution) into the aqueous phase, Eudragit S dissolved in ethanol solidified in fibre-like aggregates. It is documented that when the diffusion rate of the solvent out of the emulsion droplet was too slow, microspheres coalesced together. Conversely, when the diffusion rate of the solvent is too fast, the solvent may diffuse into the aqueous phase before stable emulsion droplets are developed, causing the aggregation of embryonic microsphere droplets.

The ratio of dichloromethane with ethanol also affected the morphology of the microballoons and the best result with a spherical shape was obtained when the ratio of ethanol to dichloromethane was 2:1. However, the average particle size increased and the wall thickness also increased as the amount of Eudragit S and ethyl cellulose increased. As the rotation speed of the propeller increased from 250 to 1000 rpm, the average particle size decreased, while maintaining its morphology. The optimum rotation speed for this experimental system was 1000 rpm, as judged from the results of particle size and size distribution, and drug content. The results are shown in Table 3.

The mean particle sizes were 110 µm for F5 microballoons and 142, 184, 435, 512, 614, and 789 µm for the formulation containing HPMC E5 in the range of 50–250 mg. The particle size of the formulation pure drug was found to be 184 µm. The compressibility index ranged between 11.41% to 14.98%. This low value of CI reveals that all the formulations showed good flowability. It was further evidenced by the low angle of repose numbers which ranged between 21.32% to 24.54%. The better flow property indicates that the floating microballoons produced were non-aggregated. HPMC E5-based Eudragit and ethyl cellullose microballoons were predominantly spherical in appearance.

The formulations F1–F4 floated with a floating lag time of 1–35 min and continued to float throughout a duration of up to 12 hr. The formulation F5 floated with a minimum floating time of 15 sec and continued to float throughout a duration of up to 12 hr.

**Fig. 1:**
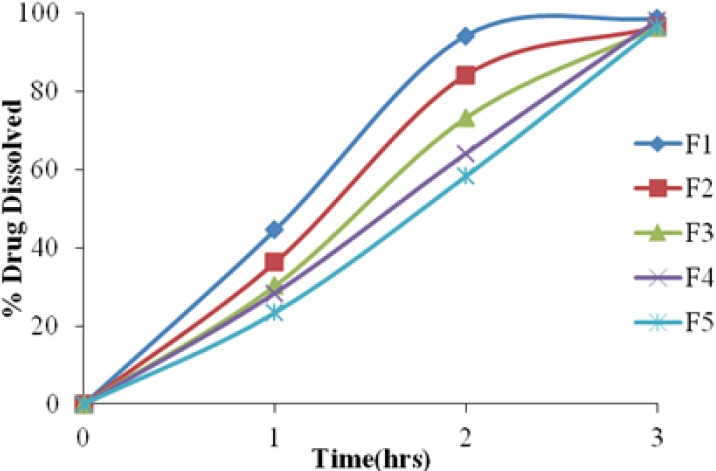
Drug release profile of stavudine granular pellets

**Fig. 2:**
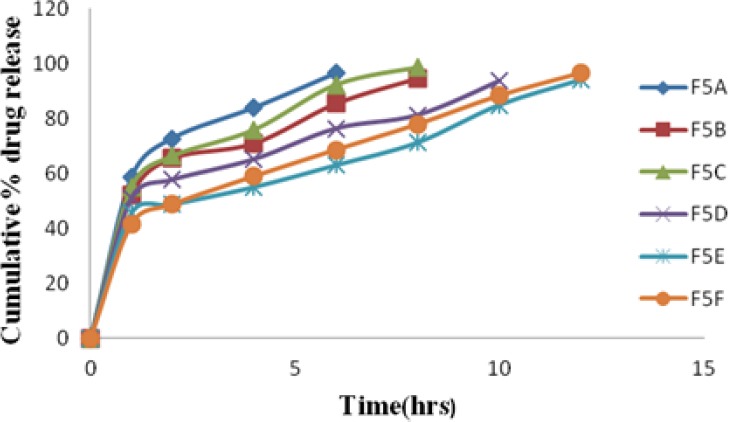
Drug release profile of floating microballoons

Dissolution studies were initially performed on the LDG and then on all the microparticle formulations by using the USP Paddle Method (Apparatus II). The drug release from the LDG was extended up to 3 hr. Among the formulations prepared, formulation F5 was further selected for preparing the microballoons. The drug release from the microballoon formulations was extended up to 12 hr in the formulations F5E–F5F, containing Eudragit RS 100 and ethyl cellulose, respectively, as rate controlling polymers. The formulations F5A–F5D, prepared by using Eudragit S 100 and ethyl cellulose at the same concentrations as rate-controlling polymers, have failed to extend drug release up to 12 hr. The results are shown in Figures 1 & 2.

All the floating microballoon formulations were found to be linear with a first-order release rate with R^2^ values in the range of 0.93–0.99. The-zero order plots for all the microparticle formulations were not linear. Thus, the rates of drug release from all the microparticle formulations were concentration-dependent and linear with first-order release rate constants (K_1_). The Higuchi constants for all the floating microparticle formulations, except for F5A–F5F, were in the range of 10–13 mg indicating the controlled drug release from the dosage form. The amount of drug released vs. time plots were found to be linear with R^2^ values in the range of 0.91–0.99. The drug release from the microballoons was by the diffusion process.

**Tab. 5:**
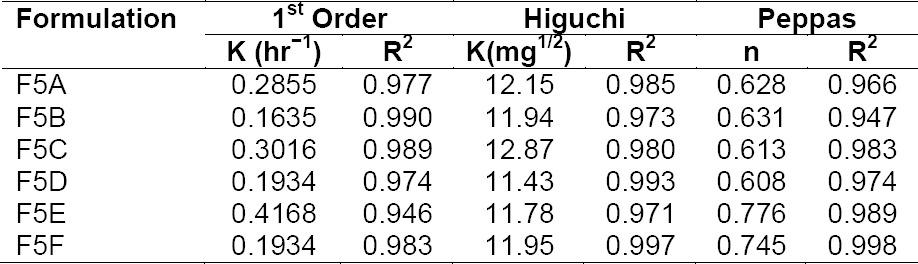
Drug release profile of stavudine controlled release floating microballoons

**Fig. 3:**
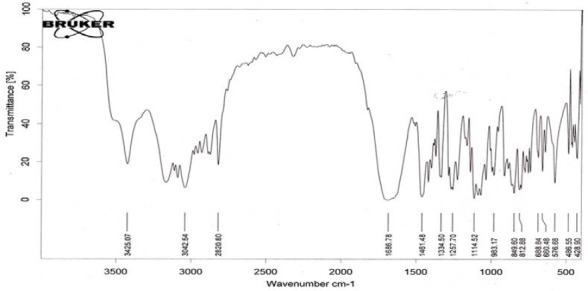
FTIR spectrum of stavudine

**Fig. 4:**
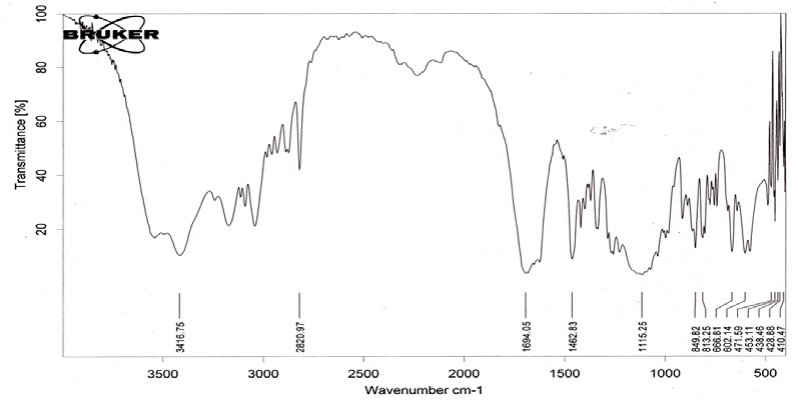
FTIR spectrum of formulation F5E

**Fig. 5:**
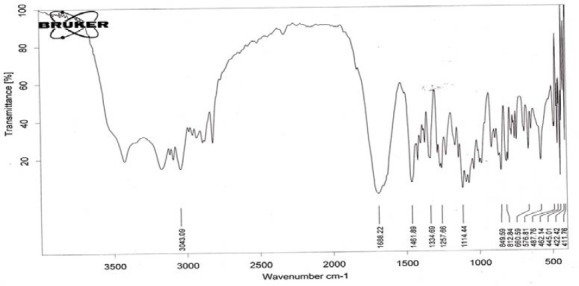
FTIR spectrum of formulation F5F

FTIR spectral studies were performed on some selected formulations of stavudine to study any drug-excipient interactions. FTIR spectral studies were performed on the BRUKER FTIR Spectrophotometer. The FTIR spectra of the pure drug of stavudine were taken initially to check the basic functional groups present in them. The spectra of stavudine pure drugs and various matrix tablet formulations are shown in Figures 3–5, respectively. The spectral studies of the stavudine formulation exhibited no more changes in the principle peaks and all the peaks were observed at specific wave numbers as that of their respective pure drugs. The characteristic OH stretching, NH stretching of a secondary amine, C-H stretching, and C=O stretching of the pure drug was unchanged in the case of the microballoons. Thus, these studies indicated that there were no major interactions between the drug, polymers, and excipients incorporated in the microballoons.

**Fig. 6:**
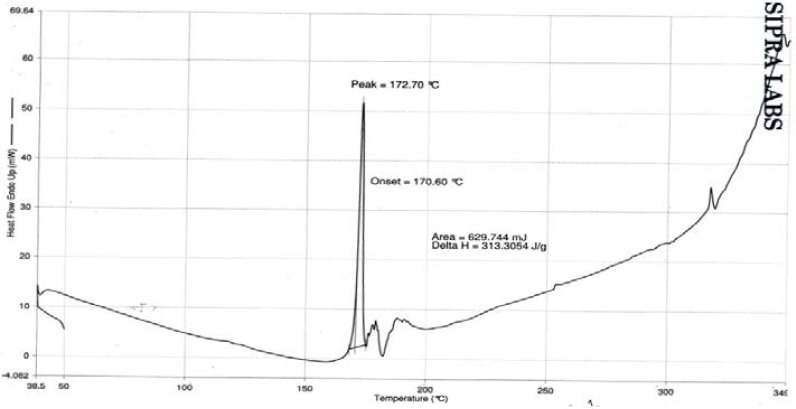
DSC spectrum of stavudine

**Fig. 7:**
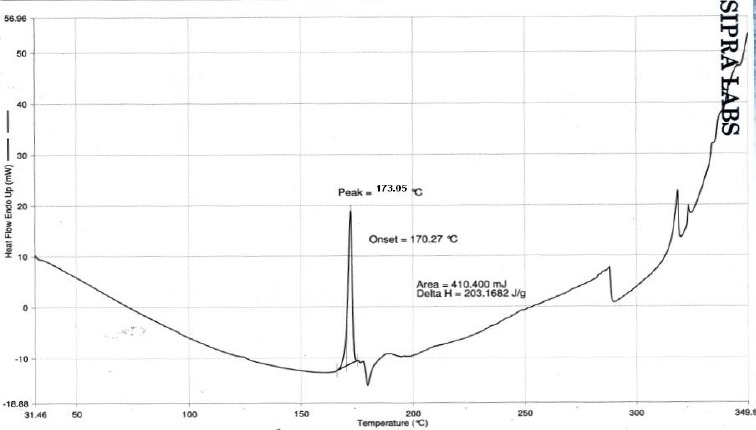
DSC spectrum of stavudine formulation F5E

**Fig. 8:**
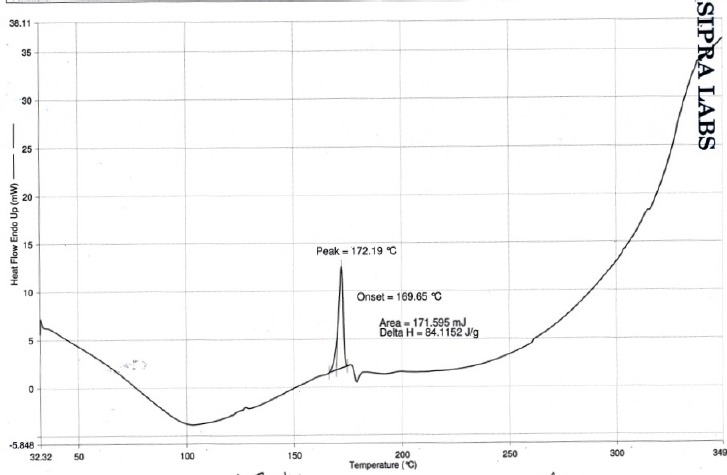
DSC spectrum of stavudine formulation F5F

DSC studies on stavudine and the optimized formulation were carried out to study the interaction between the drug and excipients used and the results of the study are shown in Figures 6 to 8. The DSC thermogram of stavudine showed a sharp endothermic peak at 172.70°C which corresponds to its melting point. The DSC thermograms of the optimized formulations F5E and F5F showed sharp endothermic peaks for stavudine at the temperatures 173.05°C and 172.19°C, respectively, without any abrupt changes in the peaks. This indicates that there were no drug-excipient interactions in the formulations.

## Conclusion

Stavudine controlled release floating microballoons were prepared by the solvent evaporation process. All the microballoons were prepared under identical conditions to minimize the processing variables. The solvent evaporation method was suitable for the drugs and polymers. The microballoon formulations prepared with the polymers Eudragit S 100 and ethyl cellulose were found to be suitable for extending drug release up to 12 hr. As the amount of polymer in the microparticle formulation increased, the drug release increased and at the same time, the floating lag time decreased. All the designed formulations of stavudine displayed first-order release kinetics, and drug release followed a non-Fickian diffusion mechanism. The formulations F5E and F5F can be considered as promising controlled release floating microballoons of stavudine.

## Authors’ Statement

### Competing Interests

The authors declare no conflict of interest.

## References

[1] Ghosh A, Nayak UK, Roy P (2007). Development, Evaluation and Method selection for the Preparation of lamivudine microspheres. Pharm Online Int J Pharm.

[2] Dev A, Binulal NS, Anitha A, Nair SV, Furuike T, Tamura H (2010). Preparation of poly (lactic acid)/chitosan nanoparticles for anti-HIV drug delivery applications. Carbohydrate Polymers.

[3] Gholap SB, Banarjee SK, Gaikwad DD, Jadhav SL, Thorat RM, Hollow, Burgess DJ, Hickey AJ, Swarbrick J, Boylan JC (1995). Microsphere technology and applications. Encyclopedia of pharmaceutical technology.

[4] Raymond C R, Sheskey PJ, Owen SC (2005). Hand book of pharmaceutical excipients.

[5] Sahoo SK, Mallick AA, Barik BB, Senapati PC (2005). Formulation and in vitro evaluation of eudragit microspheres of stavudine. Trop J Pharm Res.

[6] Behera BC, Sahoo SK, Dhal S, Barik BB, Gupta BK (2008). Characterization of glipizide-loaded polymethacrylate microspheres prepared by an emulsion solvent evaporation method. Trop J Pharm Res.

[7] Gholap SB, Banarjee SK, Gaikwad DD, Jadhav SL, Thorat RM (2010). Hollow Microsphere: A Review. Int J Pharm Sci Rev Res.

[8] Pachuau L, Sarkar S, Mazumder B (2008). Formulation and evaluation of matrix microspheres for simultaneous delivery of salbutamol sulphate and theophylline. Trop J Pharm Res.

[9] Ko JA, Park HJ, Hwang SJ, Park JB, Lee JS (2002). Preparation and characterization of chitosan microparticles intended for controlled drug delivery. Int J Pharm.

